# Increased Utilization of Overtime and Agency Nurses and Patient Safety

**DOI:** 10.1001/jamanetworkopen.2025.2875

**Published:** 2025-04-02

**Authors:** Patricia Pittman, Hong-Lun Tiunn, Qian Luo, Michael Herron, Drew Tatum, John Martin

**Affiliations:** 1 Fitzhugh Mullan Institute for Health Workforce Equity, The George Washington University, Washington, DC; 2 Premier Healthcare Alliance, Charlotte, North Carolina; 3 Premier Inc, Charlotte, North Carolina

## Abstract

**Importance:**

The use of nurse overtime and agency nurses has increased in recent years, making it important to understand the opportunities and limits of these alternative staffing strategies on patient safety outcomes.

**Objective:**

To examine the association between overtime and agency nurse staffing hours and hospital patient safety.

**Design, Setting, and Participants:**

This quality improvement study used data from 70 US hospitals, from January 2019 through December 2022. Participants included patients and nurse staff across the 70 hospitals.

**Exposure:**

The use of overtime and agency nursing.

**Main Outcomes and Measures:**

The main outcomes were 10 of the US Agency for Healthcare Research and Quality’s patient safety indicators and their associations with regular, overtime, and agency nursing hours per patient day: pressure ulcers, iatrogenic pneumothorax, in-hospital fall with hip fracture, perioperative hemorrhage or hematoma, postoperative acute kidney injury requiring dialysis, postoperative respiratory failure, perioperative thromboembolism, postoperative sepsis, postoperative wound dehiscence, and accidental puncture or laceration. A baseline Poisson regression model and a structural breakpoint analysis were used to identify safety thresholds. Pairwise interactions between staffing intensity variables and hospital bed size were also included. Adjustments were made for the COVID-19 hospital census, patient case mix, and hospital characteristics.

**Results:**

The sample included 46 hospitals in the South, 20 in the West, and 4 in the Northeast; 48 hospitals were urban, 32 hospitals had fewer than 200 beds, 26 had between 200 and 399 beds, and 12 had 400 or more beds. The average use of nurse overtime and agency nurse hours exceeded safe thresholds for pressure ulcers by 140.0% for agency staffing and by 63.6% for overtime, representing a 6.44% increase associated with excess agency nurse hours and a 2.09% increase for excess use of overtime. There also was a statistically significant association of agency hours with postsurgery hemorrhage or hematoma rates, but no breakpoint threshold. There were no significant associations with other outcomes.

**Conclusions and Relevance:**

These findings suggest that both nurse overtime and nurse agency hours are associated with increased rates of pressure ulcers, a measure that is one of the most sensitive to nursing care. In future research, hospitals could use their own data to track safe thresholds.

## Key Points

**Question:**

Are overtime and agency nurse staffing hours associated with patient safety?

**Findings:**

This quality improvement study of 70 US hospitals conducted from 2019 to 2022 found that agency nurse hours were significantly associated with negative outcomes for pressure ulcers and perioperative hemorrhage or hematoma. Exceeding breakpoint thresholds was associated with a 6.44% increase in pressure ulcers for agency nurse hours and a 2.09% increase for overtime hours; the average use exceeded these thresholds by 140.0% for agency nurse hours and by 63.6% for overtime.

**Meaning:**

These findings suggest that excessive use of overtime and agency nurse hours is associated with increased rates of pressure ulcers; future nurse staffing and patient safety research should consider disaggregating overtime and agency nurse hours from overall staffing measures so that hospitals can create their own breakpoint analyses to optimize patient safety.

## Teaser

This quality improvement study examines the association between overtime and agency nurse staffing hours and hospital patient safety at 70 US hospitals from 2019 to 2022.

## Introduction

The literature on the associations of overall nurse staffing levels with certain patient safety measures is robust. We know that overall levels of nurse staffing are associated with patient mortality,^1,2,3^ hospital-acquired central line–associated bloodstream infections,^4^ pneumonia,^5,6^ falls,^7,8,9^ and pressure ulcers.^10^ Little is known, however, about the outcomes of overtime and agency hours as variables in nurse staffing configuration research. This is important because, in recent years, the use of both overtime and agency nurses has grown exponentially, with 1 study^11^ finding that during 2020 to 2022, there was a doubling of agency and temporary labor and a 52% increase in overtime hours compared with a prepandemic baseline. A few studies have been conducted on overtime nurse hours and, using cross-sectional designs and linear modeling, found that there is a negative association with outcomes, specifically mortality,^12^ heightened risk of hospital-acquired infections (eg, central line–associated bloodstream infection^13^ and nosocomial infections^14^), elevated incidence of patient falls,^15^ and higher pressure ulcers.^16^

The association of agency nurses with patient outcomes has been far less studied, and results are mixed. A cross-sectional study by Aiken and colleagues^17^ conducted in 2013 that included 665 hospitals in 4 states concluded that higher use of agency nurses did not detrimentally affect patient outcomes after controlling for hospital characteristics. In an earlier study, Aydin and colleagues^18^ found an association between higher use of agency staff and pressure ulcers, using data from 789 medical-surgical units in 215 hospitals in 2009. However, that study was limited to California and used a convenience sample and data from a single year. Ferguson and colleagues^19^ identified an association between the prevalence of pressure ulcers and hospitals with the highest percentages of agency nurses, utilizing a sample of 605 nursing units across 166 hospitals, adjusting for patient days, and using cluster analysis across diverse hospital settings. Polancich and colleagues^20^ examined only 1 hospital and found that higher use of agency nurses was associated with longer average length of stay and higher rates of pressure ulcers from April 2021 to March 2022. In England, 2 studies^21,22^ found that an increase in the use of agency nurses was associated with higher patient mortality and unmet care needs.

The mixed findings of the US research on agency hours and the use of cross-sectional designs for both agency and overtime research suggest the need for a more comprehensive and methodologically rigorous examination of the association of both agency and overtime hours with patient outcomes.^23^ This study explores whether there are safe thresholds for overtime and agency nurse hours beyond which patient safety may decline. Going beyond a linear regression to identify the dose effect of these 2 supplementary nurse staffing strategies is important because some use of overtime and agency nurses may be necessary, especially during public health emergencies and seasonal surges in patient demand. Thus, this study examines the association of overtime and agency nursing hours with 10 US Agency for Healthcare Research and Quality patient safety measures: pressure ulcer (PSI-3), iatrogenic pneumothorax (PSI-6), in-hospital fall with hip fracture (PSI-8), perioperative hemorrhage or hematoma (PSI-9), postoperative acute kidney injury requiring dialysis (PSI-10), postoperative respiratory failure (PSI-11), perioperative pulmonary embolism or deep vein thrombosis (PSI-12), postoperative sepsis (PSI-13), postoperative wound dehiscence (PSI-14), and accidental puncture or laceration (PSI-15) during the 4 years 2019 through 2022.

## Methods

### Data and Sample

In this quality improvement study, we used a proprietary research database maintained by Premier, Inc, comprising daily reports on utilization and quality outcomes, as well as biweekly payroll-based reports on staffing from 70 hospitals that are Premier members and that granted permission for their deidentified data to be used for research. We used data from January 2019 to December 2022. This study used deidentified secondary data and did not require institutional review board approval, in accordance with 45 CFR §46. The study followed the Standards for Quality Improvement Reporting Excellence (SQUIRE) reporting guidelines.^24^

Given the inherently rare nature of hospital adverse events, a challenge in conducting such research is obtaining a dataset with sufficient variability. To address this, we aggregated all data to the level of hospital calendar quarters. The hospital-quarter panel data allow for meaningful variation in quality outcomes cross-sectionally and longitudinally. The panel data enabled us to isolate the impact of varying nurse staffing levels and composition on outcomes while controlling for the caseload and case mixes.

### Variables

The primary outcomes of our study were PSI-3, PSI-6, PSI-8, PSI-9, PSI-10, PSI-11, PSI-12, PSI-13, PSI-14, and PSI-15. Premier members constructed measures according to guidelines provided by the Agency for Healthcare Research and Quality.^25^

The independent variables of interest related to nurse staffing levels and types. To examine the impact of the mix of the kinds of nurse workforce hours and the intensity of nurse staffing, we analyzed the regular and overtime hours by full-time Registered Nurses (RNs) and Licensed Practical Nurses (LPNs), measured in nursing hours per patient day (HPPD). We included LPNs because, as with most hospitals, there were very few in this sample, and where they do exist, they substantially overlap roles with RNs. Hospitals in the sample provided data to Premier’s detailed payroll-based staffing database, allowing us to track how this sample of hospitals deployed their nurse staffing from 2019 through 2022. This includes 1 year before the COVID-19 pandemic, the first 2 years of the pandemic, and 1 year following the acute phase of the pandemic.

In addition to the primary independent variables, we controlled for nurse assistive personnel (NAP) HPPD, which research suggests is critical to determining nurse staffing outcomes.^23,26^ We also included the number of acute beds as a proxy measure for hospital capital.^27,28^ This variable was key to assessing how a hospital’s size and resource availability might influence patient outcomes. To account for patients with COVID-19, which introduced high-acuity levels and workflow changes, we included the hospital COVID-19 census.

Finally, we introduced a set of dummy variables to account for variation in the temporal impact of the COVID-19 pandemic on nurse staffing patterns and patient outcomes since patient acuity and workflows change over time. We delineated 1 pre–COVID-19 pandemic phase, spanning from January 1, 2019, to March 31, 2020, and 3 distinct COVID-19 pandemic waves as follows: April 1, 2020, to September 30, 2020; October 1, 2020, to March 31, 2021; and April 1, 2021, to December 31, 2022.

### Statistical Analysis

We first used a hierarchical Poisson regression model for all 10 PSIs to analyze the association between nurse staffing and patient outcomes. The observed incidences of PSIs were the dependent variable of the regression. This approach transforms the Poisson regression model into a rate regression, focusing on the frequency of observed occurrences relative to the expected counts. The model accounted for variations in the underlying patient risk profiles by using expected incidences as the offset variable. This adjustment was key, considering that hospitals with more patients or patients with higher risk factors might naturally expect a greater number of incidences. This variation occurs independently of the nurse staffing levels, and the model’s adjustment aims to distinguish the influence of nurse staffing levels from inherent risk factors.

We introduced all independent variables as linear terms in the baseline regression model (eAppendix 1 in Supplement 1). These included various nursing staff variables in each hospital *i* during each quarter *t*, such as RN or LPN regular, overtime, and agency HPPD. In addition, we accounted for the interactions between the percentage of hospital census attributed to COVID-19 and the distinct COVID-19 waves. This approach enabled us to assess the direct linear associations of these variables and their dynamic interactions within the context of the pandemic.

To capture potential nonlinear relationships between nurse staffing intensity and patient outcomes, we identified structural breakpoints for all 3 types of nursing hours (see eAppendix 2 in Supplement 1). Structural breakpoint analysis, which is commonly used in economics to detect changes in regression coefficients following external events,^29,30^ has also been applied in health services research to assess policy impacts on quality of care.^31^ We conducted a grid search of possible breakpoints across percentiles of each nursing hours distribution. A sup-Wald test then selected the breakpoint that best explained changes in outcomes associated with these staffing levels. Each breakpoint entered our regression model through 2 mechanisms: a shift in the level of association and a modification in the slope, reflecting both an intercept change and an altered rate of association between staffing intensity and patient outcomes.

To capture potential nonlinear associations more comprehensively, we also applied spline function models, which provide increased flexibility over traditional approaches. Schuster and colleagues^32^ advocate for spline functions, including B-splines, as effective tools for modeling complex patterns in the data, free from the limitations of polynomial terms or categorical grouping. Using B-splines on our nurse staffing intensity variables allowed us to uncover nuanced patterns and potential thresholds that might be overlooked in linear and breakpoint models. Data were analyzed using R statistical software version 4.1.3 (R Project for Statistical Computing). Two-sided *P* < .05 is considered statistically significant.

## Results

Overall, our sample of 70 hospitals reflects a reasonable national distribution, although it remains a convenience sample. Thirty-two hospitals (45.7%) had a total bed count of 0 to 199, followed by 26 (37.1%) with 200 to 399 beds, and 12 (17.1%) with 400 or more beds. Regionally, 46 hospitals (65.7%) were located in the South, with 23.9% of Southern hospitals having 400 or more beds. In contrast, among 20 hospitals in the West, 12 (60.0%) had 0 to 199 beds, and among 4 hospitals (5.7% of the total) in the Northeast, 3 hospitals (75.0%) had fewer than 200 acute beds. In terms of urban vs rural settings, most hospitals were urban (48 hospitals [68.6%]), particularly in the Northeast (4 hospitals [100.0%]) and West (12 hospitals [60.0%]). Conversely, rural hospitals were most common in the South (14 hospitals [30.4%]). Most hospitals were nonteaching (64 hospitals [91.4%]), with the few teaching hospitals (6 hospitals [8.6%]) located exclusively in the South (Table 1).

**Table 1.** Distribution of Hospital Characteristics by Region^a^

Characteristic	Hospitals, No. (%)			
	South (n = 46 [65.7%])	Northeast (n = 4 [5.7%])	West (n = 20 [28.6%])	Total (N = 70 [100.0%])
Total bed count				
0-199	18 (39.1)	2 (50.0)	12 (60.0)	32 (45.7)
200-399	17 (37.0)	2 (50.0)	7 (35.0)	26 (37.1)
≥400	11 (23.9)	0	1 (5.0)	12 (17.1)
Total acute bed count				
0-199	21 (45.7)	3 (75.0)	14 (70.0)	38 (54.3)
200-399	15 (32.6)	1 (25.0)	5 (25.0)	21 (30.0)
≥400	10 (21.7)	0	1 (5.0)	11 (15.7)
Rural vs urban status				
Rural	14 (30.4)	0	8 (40.0)	22 (31.4)
Urban	32 (69.6)	4 (100.0)	12 (60.0)	48 (68.6)
Teaching status				
Nonteaching	40 (87.0)	4 (100.0)	20 (100.0)	64 (91.4)
Teaching	6 (13.0)	0	0	6 (8.6)

^a^ The regions represent US Census Bureau regions, where each region includes the following states: South: Alabama, Arkansas, Delaware, Florida, Georgia, Kentucky, Louisiana, Maryland, Mississippi, North Carolina, Oklahoma, South Carolina, Tennessee, Texas, Virginia, West Virginia, and Washington, DC; Northeast: Connecticut, Maine, Massachusetts, New Hampshire, New Jersey, New York, Pennsylvania, Rhode Island, and Vermont; and West: Alaska, Arizona, California, Colorado, Hawaii, Idaho, Montana, Nevada, New Mexico, Oregon, Utah, Washington, and Wyoming.

The mean (SD) total HPPD for RNs, LPNs, and NAP combined from 2019 to 2022 was 2.28 (1.43). Of those hours, 4.7% (mean [SD], 0.09 [0.09] hours) were attributable to overtime, and 6.0% (mean [SD], 0.09 [0.24] hours) were attributable to agency nurse hours. For RNs only, the mean (SD) total HPPD was 2.14 (1.33), with 4.5% (mean [SD], 0.09 [0.09] hours) of the hours allocated to overtime and 6.8% (mean [SD], 0.09 [0.24] hours) to agency nurse hours. For LPNs, the mean (SD) total HPPD was 0.01 (0.03). In this category, 1.7% (mean [SD], 0.00 [0.00] hours) of the hours were ascribed to overtime, and agency staff accounted for 0% (mean [SD], 0.01 [0.00] hours). The mean (SD) total HPPD for NAP was 0.12 (0.19), with 4.8% (mean [SD], 0.01 [0.01] hours) overtime and 0.1% of agency staff hours (Table 2). Some values here and in Table 2 appear as 0.00 due to rounding; however, the corresponding proportions are not actually zero. Refer to eTable 1 in Supplement 1 for detailed descriptive statistics, including medians and IQRs.

**Table 2.** Descriptive Summary of Facility and Nursing Hours Per Patient Day Characteristics

Variable	Hours per patient day				
	All years	2019	2020	2021	2022
Total, mean (SD)					
Combined	2.28 (1.43)	2.62 (1.28)	2.80 (1.46)	1.83 (1.34)	1.76 (1.31)
RN	2.14 (1.33)	2.47 (1.16)	2.63 (1.34)	1.72 (1.27)	1.65 (1.25)
LPN	0.01 (0.03)	0.01 (0.04)	0.01 (0.03)	0.01 (0.02)	0.01 (0.03)
NAP	0.12 (0.19)	0.14 (0.21)	0.15 (0.24)	0.10 (0.15)	0.09 (0.15)
Normal, mean (SD) [%]					
Combined	2.09 (1.34) [89.2]	2.47 (1.18) [94.0]	2.61 (1.32) [93.3]	1.63 (1.26) [82.6]	1.57 (1.24) [86.1]
RN	1.97 (1.24) [85.1]	2.33 (1.06) [93.5]	2.45 (1.20) [92.8]	1.53 (1.20) [75.2]	1.47 (1.17) [77.1]
LPN	0.01 (0.03) [24.4]	0.01 (0.04) [20.6]	0.01 (0.03) [29.5]	0.01 (0.02) [23.8]	0.01 (0.02) [23.6]
NAP	0.12 (0.19) [66.6]	0.13 (0.20) [73.6]	0.15 (0.23) [74.0]	0.10 (0.14) [57.7]	0.09 (0.14) [59.3]
Overtime, mean (SD) [%]					
Combined	0.09 (0.09) [4.7]	0.10 (0.08) [4.3]	0.11 (0.09) [4.1]	0.09 (0.10) [5.4]	0.08 (0.09) [5.0]
RN	0.09 (0.09) [4.5]	0.09 (0.07) [4.3]	0.10 (0.08) [4.1]	0.09 (0.09) [5.1]	0.08 (0.09) [4.4]
LPN	0.00 (0.00) [1.7]	0.00 (0.00) [2.2]	0.00 (0.00) [1.6]	0.00 (0.00) [2.0]	0.00 (0.00) [0.9]
NAP	0.01 (0.01) [4.8]	0.01 (0.01) [4.3]	0.01 (0.01) [4.3]	0.00 (0.01) [4.4]	0.00 (0.01) [6.3]
Agency, mean (SD) [%]					
Combined	0.09 (0.24) [6.0]	0.05 (0.19) [1.8]	0.08 (0.31) [2.6]	0.11 (0.23) [11.6]	0.11 (0.23) [8.8]
RN	0.09 (0.24) [6.8]	0.05 (0.19) [1.9]	0.08 (0.31) [2.7]	0.11 (0.23) [13.5]	0.11 (0.23) [10.1]
LPN	0.00 (0.00) [0.0]	0.00 (0.00) [0.0]	0.00 (0.00) [0.0]	0.00 (0.00) [0.0]	0.00 (0.00) [0.0]
NAP	0.00 (0.00) [0.1]	0.00 (0.00) [0.0]	0.00 (0.00) [0.0]	0.00 (0.00) [0.1]	0.00 (0.00) [0.2]

Abbreviations: LPN, licensed practical nurse; NAP, nurse assistive personnel; RN, registered nurse.

Staffing levels and resource use showed significant variation across COVID-19 waves. RN and LPN total hours peaked in wave 1 at a mean (SD) of 2.79 (1.42) HPPD, reflecting increased demand, then declined to 1.69 (1.27) HPPD in wave 3. Overtime remained steady across waves, with a mean (SD) of 0.09 (0.09) HPPD, while agency hours surged in wave 3, reaching a high of 0.11 (0.24) HPPD. NAP hours increased in wave 1 (mean [SD], 0.17 [0.27] hours) before tapering in later waves. The mean (SD) number of acute care beds increased from 248.47 (201.37) beds before COVID-19 to a high of 258.37 (200.00) beds in wave 3. The hospital COVID-19 census increased sharply, beginning at a mean (SD) of 0.09% (0.42%) before COVID-19, which included March 2020, and peaking at 19.81% (8.59%) in wave 2. Patient safety outcomes varied notably. Pressure ulcer rates initially decreased in wave 1 but increased in waves 2 and 3 to a mean (SD) of 1.32 (3.45) cases per 1000 discharges. Postoperative pulmonary embolism or deep vein thrombosis (mean [SD], 12.90 [2.30] cases per 1000 discharges) and perioperative hemorrhage or hematoma (mean [SD], 1.72 [2.85] cases per 1000 discharges) rates were notably steady across the pre–COVID-19 period and the pandemic waves. Perioperative hemorrhage or hematoma (PSI-9) incidents remained constant at 1.27 cases per 1000 discharges, whereas iatrogenic pneumothorax (PSI-6) was relatively low at 0.27 cases per 1000 discharges. In-hospital falls with hip fracture (PSI-8) were infrequent, averaging 0.13 cases per 1000 discharges. Postoperative physiological and metabolic derangement (PSI-10) averaged 0.28 cases per 1000 discharges, whereas postoperative respiratory failure (PSI-11) had a higher rate of 1.64 cases per 1000 discharges. Rates for perioperative pulmonary embolism or deep vein thrombosis (PSI-12) remained consistent, averaging 1.68 cases per 1000 discharges. Postoperative sepsis (PSI-13) incidents averaged 0.93 cases per 1000 discharges. Postoperative wound dehiscence (PSI-14) maintained a low rate of 0.22 cases per 1000 discharges, while accidental puncture or laceration (PSI-15) was slightly higher at 0.41 cases per 1000 discharges. These findings provide a comprehensive overview of patient safety outcomes across the all-time period (Table 3).

**Table 3.** Independent and Outcome Variables Across COVID-19 Phases

Variables	Mean (SD)			
	Pre–COVID-19 (January 1, 2019, to March 31, 2020)	Wave 1 (April 1, 2020, to September 30, 2020)	Wave 2 (October 1, 2020, to March 31, 2021)	Wave 3 (April 1, 2021, to December 31, 2022)
Independent variables				
Hours per patient day^a^				
RN or LPN total	2.50 (1.22)	2.79 (1.42)	2.04 (1.21)	1.69 (1.27)
RN or LPN overtime	0.09 (0.08)	0.09 (0.08)	0.10 (0.09)	0.08 (0.09)
RN or LPN agency	0.06 (0.22)	0.09 (0.33)	0.08 (0.22)	0.11 (0.24)
NAP total^b^	0.14 (0.22)	0.17 (0.27)	0.11 (0.17)	0.10 (0.15)
Acute beds, No.	248.47 (201.37)	246.06 (201.60)	249.99 (202.01)	258.37 (200.00)
Hospital COVID-19 census, %	0.09 (0.42)	9.41 (8.97)	19.81 (8.59)	13.29 (9.36)
Outcome variables, No. of cases/1000 discharges				
Pressure ulcer	0.86 (1.57)	0.52 (1.18)	0.90 (1.74)	1.32 (3.45)
Perioperative hemorrhage or hematoma	1.35 (2.50)	1.13 (2.19)	1.30 (2.28)	1.29 (2.18)
Iatrogenic pneumothorax	0.29 (0.63)	0.30 (0.57)	0.22 (0.54)	0.27 (0.59)
In-hospital fall with hip fracture	0.12 (0.36)	0.11 (0.31)	0.16 (0.40)	0.13 (0.38)
Postoperative physiologic and metabolic derangement	0.39 (0.90)	0.21 (0.55)	0.24 (0.60)	0.27 (0.69)
Postoperative respiratory failure	2.04 (3.84)	1.43 (2.77)	1.57 (2.66)	1.53 (2.82)
Perioperative pulmonary embolism or deep vein thrombosis	1.67 (2.78)	1.59 (2.50)	1.60 (2.86)	1.84 (3.01)
Postoperative sepsis	1.01 (2.05)	0.81 (1.81)	0.96 (1.96)	0.95 (1.93)
Postoperative wound dehiscence	0.23 (0.58)	0.25 (0.63)	0.18 (0.60)	0.23 (0.70)
Accidental puncture or laceration	0.35 (0.81)	0.35 (0.85)	0.39 (0.82)	0.55 (1.10)

Abbreviations: LPN, licensed practical nurse; NAP, nurse assistive personnel; RN, registered nurse.

^a^ These data are the summation of RN and LPN total, overtime, and agency hours per patient day.

^b^ NAP total hours per patient day encompasses the sum of normal, overtime, and agency hours per patient day for NAPs.

In our baseline regression models (Table 4), after adjusting for other variables, we found that for every 10% increase in RN or LPN agency HPPD (mean [SD], 0.14 [0.22] hours) was associated with a 0.79% higher risk of pressure ulcers (incidence rate ratio [IRR], 1.80; 95% CI, 1.16-2.78; *P* = .008). For each 10% increase in RN or LPN overtime HPPD (with a mean [SD] of 0.09 [0.09] hours), there was an associated 0.73% higher risk of pressure ulcers, but the IRR of 2.26 was not statistically significant (95% CI, 0.55-9.30; *P* = .25). For postoperative hemorrhage or hematoma, a 10% increase in RN or LPN agency HPPD was associated with a 0.38% higher risk (IRR, 1.46; 95% CI, 1.02-2.08; *P* = .03). In contrast, RN or LPN overtime HPPD was not significantly associated with postoperative hemorrhage or hematoma (IRR, 1.38; 95% CI, 0.42-4.47; *P* = .59). We found no other PSI with statistically significant associations (eTable 2 in Supplement 1).

**Table 4.** Baseline Regression Model Outcomes on Primary Outcomes in Hospitals^a^

Variable	Pressure ulcers		Postoperative hemorrhage or hematoma	
	IRR (95% CI)	*P* value	IRR (95% CI)	*P* value
RN or LPN work HPPD	0.96 (0.85-1.1)	.62	0.99 (0.9-1.1)	.90
RN or LPN overtime HPPD	2.26 (0.55-9.30)	.25	1.38 (0.42-4.47)	.59
RN or LPN agency HPPD	1.80 (1.16-2.78)	.008	1.46 (1.02-2.08)	.03
NAP work HPPD	2.75 (1.51-5.01)	<.001	0.61 (0.33-1.12)	.11
Intercept	0.45 (0.3-0.67)	<.001	1.03 (0.77-1.37)	.86

Abbreviations: HPPD, hours per patient per day; IRR, incidence rate ratio; LPN, licensed practical nurse; NAP, nurse assistive personnel; RN, registered nurse.

^a^ This table shows coefficient estimates obtained from our baseline regression model that control for NAP total HPPD, hospital acute beds, percentage of the hospital census with COVID-19, COVID-19 waves, and the interactions between the percentage of hospital census attributed to COVID-19 and the distinct COVID-19 waves. Covariates include number of hospital beds, percentage hospital census with COVID-19, COVID-19 waves, and the interactions between percentage of patients with COVID-19 and COVID-19 waves.

We conducted the structural breakpoint model for the 2 outcomes that were significant but found a breakpoint for pressure ulcers only (Table 5). Specifically, breakpoints were observed at 0.090 HPPD for RN or LPN agency HPPD and 0.055 HPPD for RN or LPN overtime HPPD. Outside the identified breakpoints, RN or LPN agency HPPD showed a positive association with pressure ulcer risk, with an IRR of 1.44, while RN or LPN overtime HPPD had an IRR of 1.40, associated with higher risk at every 10% increase of agency staffing hours (0.46%) and overtime (0.33%). Because of the absence of a statistically significant breakpoint for PSI-9, findings related to PSI-9 are not included.

**Table 5.** Structural Break Point Regression Model Outcomes on Pressure Ulcers in Hospitals^a^

Variable	Break point identified	*P* value
RN or LPN agency HPPD	0.090	NA
RN or LPN overtime HPPD	0.055	NA
Estimate (SE)		
Agency HPPD breakpoint	−6.32 (2.06)	.002
Overtime HPPD breakpoint	−15.95 (4.49)	<.001
RN or LPN agency HPPD	6.69 (2.04)	.001
RN or LPN overtime HPPD	16.29 (4.52)	<.001
RN or LPN work HPPD	−0.15 (0.08)	.07
NAP work HPPD	1.14 (0.32)	<.001
Intercept	−1.23 (0.24)	<.001

Abbreviations: HPPD, hours per patient per day; LPN, licensed practical nurse; NA, not applicable; NAP, nurse assistive personnel; RN, registered nurse.

^a^ This table presents coefficient estimates from a structural break point regression model analyzing outcomes related to pressure ulcers in hospitals. The model controls for various HPPD metrics, including RN or LPN agency HPPD and RN or LPN overtime HPPD, with identified break points at 0.090 and 0.055, respectively. Additional controls include agency HPPD and overtime HPPD breakpoints, RN or LPN work HPPD, and NAP work HPPD, along with an intercept and other covariates. Robust SEs are shown in parentheses. Covariates include number of hospital beds, percentage hospital census being patients with COVID-19, COVID-19 waves, and the interactions between percentage of patients with COVID-19 and COVID-19 waves.

Thus, with a mean use of agency nurse hours at 0.14 HPPD, we found that sample hospitals were 140% over the safe threshold identified, representing a 6.44% increased risk of pressure ulcers. At a mean of 0.09 HPPD of overtime nurse hours, hospitals were 63.6% over the estimated safe threshold identified in our structural breakpoint model, translating to a 2.09% increased risk of pressure ulcers among patients due to excess use of overtime.

As a robustness check for pressure ulcers, we applied spline function models (eTable 3 in Supplement 1) to further examine RN or LPN agency HPPD and overtime HPPD, incorporating threshold effects similar to those in structural breakpoint models. In these spline models, RN or LPN agency hours initially demonstrated a protective association with lower pressure ulcer risk. Still, this association reversed at higher agency hours. Likewise, RN or LPN overtime hours initially showed a protective effect, with higher overtime correlating with an increased risk of pressure ulcers once a certain threshold was exceeded. Results of nonlinear regression models are shown in eFigure 1 and eFigure 2 in Supplement 1.

## Discussion

In this quality improvement study, the results of our linear model suggest that of the 10 PSIs, only 2 showed a significant negative association with agency hours: PSI-3 (pressure ulcer) and PSI-9 (perioperative hemorrhage or hematoma). Only pressure ulcer rates were significant for overtime hours. Both of these measures are deemed nurse-sensitive, meaning that prior studies have shown an association with overall nursing levels. Therefore, changes in the configuration of nurses could be a plausible mechanism. On the other hand, we were surprised, in particular, not to find an association with PDI-11, failure to rescue, which was both a relatively frequent occurrence in our sample (making it easier to detect in the model), and also a nurse-sensitive measure. This may be related to how the overtime and agency nurses were distributed by unit, but there is no way to determine this in our study, given the aggregation of units in this analysis.

Importantly, the structural breakpoint analysis allows us to see beyond the initial conclusion of a small effect for agency and overtime hours. These findings suggest that surpassing specified HPPD values of agency and overtime hours can precipitate a shift in the risk trend of pressure ulcers, transitioning from a decrease to an increase in risk as these types of HPPD levels continue to ascend.

Pressure ulcers are a widely acknowledged measure of patient safety in hospital settings and are sensitive to overall nurse staffing levels.^6^ They are among the measures most studied and most sensitive to nursing care, as documented in systematic reviews.^33^ Annually, the health care system in the US contends with approximately 2.5 million pressure ulcer cases,^34^ resulting in as many as 60 000 deaths^35^ and an additional cost of $72 000 for all payers^36^ and $43 000 for Medicare^37^ per stay associated with pressure ulcers. Given the preventable nature of pressure ulcers, the Centers for Medicare & Medicaid Services enacted policies in 2008 that targeted a reduction in reimbursements for treatments related to severe pressure ulcers (identified as stage 3, stage 4, and unstageable).^38^ This policy signified a concerted effort to promote the adoption of more effective preventive measures, including, presumably, adequate nurse staffing levels. Our findings suggest that a better understanding of how the total nurse staffing hours are constructed across regular, overtime, and agency hours is also relevant.

The findings also add to prior research on the effects of agency nurse hours. Although the findings are consistent with some of the prior studies,^19,20,21^ they conflict with the early work of Aiken and colleagues.^17^ This may be a function of the dose of agency dependency on agency nurses in 2013 was far lower than during the pandemic and even today.^17^ Finally, our findings on overtime add nuance to prior research, identifying the lower levels at which overtime hours can reduce pressure ulcers, as well as the threshold beyond which the incidence increases.

In summary, given that the use of both overtime and agency nurse hours is likely necessary during a national public health crisis, and perhaps even during normal seasonal fluctuations in patient demand, the question is how much can hospitals rely on these backup strategies before they risk endangering patient outcomes? This study provides a first glimpse into this issue, acknowledging both the benefits of overtime and agency nurses, as well as the dangers when they are overused in the case of pressure ulcers.

The hotly debated policy question of the day is what role state and federal payers and regulators could play in incentivizing hospitals to prioritize regular staff over alternative staffing strategies. Some states prohibit mandatory overtime, and the American Nurses Association now advocates for Federal (or state) legislative guardrails on overall staffing levels^39^ that is akin to the 2002 California and the 2023 Oregon nurse staffing mandates.^40^ Others contend that the Centers for Medicare & Medicaid Services should mandate minimum staffing levels as a prerequisite for licensing hospitals receiving Medicare payments, mirroring the approach already implemented for nursing homes.^41^ Finally, some states require public reporting of nurse staffing levels, although there is no evidence that this approach has improved staffing.^42^

### Limitations

There are several limitations to this study. First, we use a convenience sample that cannot be generalized to all US hospitals. Premier-affiliated hospitals providing data used for this study are typically larger and better financed than the average US hospital. The findings are, therefore, not generalizable, especially to rural, critical access hospitals. In response to this, our analytical strategy included the integration of risk-adjusted patient outcome measures, and the adoption of hospital bed count as a surrogate for institutional size, aiming to offset the skewed representation of hospital types and severity of patients within our sample.

Second, our effort to document and analyze the distribution of floating staff across various departmental units encountered major obstacles, primarily due to the lack of uniform reporting standards across the facilities. This inconsistency restricted our capability to include charge nurses, who may assist with patient care during COVID-19 and other shortage times. Given that charge nurses are regular staff, if this limitation impacted findings, it would likely have been by lowering the average uses of agency nurses, a difference that would not likely impact our overall conclusions.

Third, although our model accounted for the diverse categories of staffing hours (regular, overtime, and agency), it did not specifically explore the potential for substitution among these different staffing modalities. In future research, we plan to address this gap, offering a more nuanced exploration of how staffing configurations impact hospital operations and patient care outcomes.

Fourth, the data used in this study were from a unique time frame in the US in that much of the period covered was during the COVID-19 pandemic. The advantage of this was that it provided a natural experiment (ie, fluctuations in staffing) utilized in this project. However, we recognize that 3 of the 4 years included were not representative of normal hospital operations. We did attempt to control for external factors that could have been associated with the outcome and COVID-19 itself in order to mitigate any influence they may have had.

## Conclusions

This study suggests that regardless of the policy solution to overall nurse staffing levels, it is important to recognize that overtime and agency nurse hours may not have the same value as regular nurse staff for certain outcomes. As such, where possible, differentiated reporting requirements should be explored. Such transparency measures may not be used by the general public, but payers and advocacy groups could use the data to evaluate hospitals and refine financial incentives. Hospitals could also create their own analyses and establish breakpoints for overtime and agency nurse hours to maximize patient safety.

## Notes

Supplement 1. **eAppendix 1.** Equation 1: Baseline regression model **eAppendix 2.** Equation 2: Model specification using structural break point regression model **eTable 1.** Detailed descriptive statistics **eTable 2.** Baseline regression model for primary outcomes with healthcare personnel hours per patient day variables in hospitals **eTable 3.** Baseline regression model for primary outcomes with healthcare personnel hours per patient day variables in hospitals (cont.) **eFigure 1.** Non-linear regression model outcomes for RN/LPN overtime HPPD on the PSI-03 in hospitals **eFigure 2.** Non-linear regression model outcomes for RN/LPN agency HPPD on PSI-03 in hospitals

Supplement 2.
Data Sharing Statement
