# Supplementary material for: Increased Utilization of Overtime and Agency Nurses and Patient Safety
Source: JAMA Netw Open. 2025 Apr 2;8(4):e252875. doi: 10.1001/jamanetworkopen.2025.2875 (PMC11966309; doi:10.1001/jamanetworkopen.2025.2875)
Supplement: Supplement 1. — eAppendix 1. Equation 1: Baseline regression model eAppendix 2. Equation 2: Model specification using structural break point regression model eTable 1. Detailed descriptive statistics eTable 2. Baseline regression model for primary outcomes with healthcare personnel hours per patient day variables in hospitals eTable 3. Baseline regression model for primary outcomes with healthcare personnel hours per patient day variables in hospitals (cont.) eFigure 1. Non-linear regression model outcomes for RN/LPN overtime HPPD on the PSI-03 in hospitals eFigure 2. Non-linear regression model outcomes for RN/LPN agency HPPD on PSI-03 in hospitals [file jamanetwopen-e252875-s001.pdf]

Supplemental Online Content

Pittman P, Tiunn HL, Luo QE, Herron M, Tatum D, Martin J. Increased utilization of overtime and agency nurses and patient safety. *JAMA Netw Open*. 2025;8(4):e252875. doi:10.1001/jamanetworkopen.2025.2875

eAppendix 1. Equation 1: Baseline regression model

eAppendix 2. Equation 2: Model specification using structural break point regression model

eTable 1. Detailed descriptive statistics

eTable 2. Baseline regression model for primary outcomes with healthcare personnel hours per patient day variables in hospitals

eTable 3. Baseline regression model for primary outcomes with healthcare personnel hours per patient day variables in hospitals (cont.)

eFigure 1. Non-linear regression model outcomes for RN/LPN overtime HPPD on the PSI-03 in hospitals

eFigure 2. Non-linear regression model outcomes for RN/LPN agency HPPD on PSI-03 in hospitals

This supplemental material has been provided by the authors to give readers additional information about their work.

© 2025 Pittman P et al. *JAMA Network Open*.

eAppendix 1. Equation 1: Baseline regression model

$$\ln(E[y_{it}]) = \alpha + \Sigma_p \beta_p HPPD_{itp} + \boldsymbol{\delta_{it} X_{it}} + \sigma_i + \ln(\tilde{y}_{it})$$

where

- i indexes hospital and t indexes time in quarters, and p indexes type of nursing hours ($p \in \{regular, overtime, agency\}$).
- y_{it} is the observed PSI outcome at hospital i and quarter t.
- $\tilde{y}_{it}$ is the expected PSI outcome at hospital i and quarter t, providing an adjustment for patient case mix.
- $HPPD_{itp}$ are our primary independent variables of nursing hours per patient day by type p with β_p as the corresponding coefficient.
- $\boldsymbol{X_{it}}$ is a vector of the covariates with $\boldsymbol{\delta}$ as the associated vector of coefficients.
- σ_i is the hospital-level random effect, and α is the intercept.

© 2025 Pittman P et al. *JAMA Network Open*.

eAppendix 2. Equation 2: Model specification using structural break point regression model

$$\ln(E[y_{it}]) = \alpha + \Sigma_p \beta_p HPPD_{itp} + \Sigma_p \gamma_p Breakpoint_{itp} + \Sigma_p \phi_p \left(Breakpoint_{itp} \times HPPD_{itp}\right) + \boldsymbol{\delta X_{it}} + \sigma_i + \ln(\tilde{y}_{it})$$

where

- Definitions for $i, t, p, \alpha,$ and σ_i are the same as in Equation 1.
- β_p are the coefficients of $HPPD_{itp}$ before reaching the breakpoint threshold.
- $Breakpoint_{itp}$ are the breakpoint variables that equals 1 if nursing hours in category p exceeds the breakpoint threshold and 0 otherwise.
- γ_p indicates the level change in outcome when nursing hours in category p cross the breakpoint threshold.
- ϕ_p captures the slope change in outcome associated with $HPPD_{itp}$ when nursing hours in category p cross the breakpoint threshold.

© 2025 Pittman P et al. *JAMA Network Open*.

eTable 1. Detailed descriptive statistics

name	All years
Total, mean, median, SD, IQR	
Combined	2.275829, 2.110583, 1.425451, 1.374267
RN	2.143395, 2.017756, 1.328702, 1.297297
LPN	0.008863, 0, 0.031119, 0.000282
NAP	0.123571, 0.039243, 0.193782, 0.177979
Normal, mean, median, SD, IQR	
Combined	2.094054, 1.986699, 1.337968, 1.335751
RN	1.967461, 1.892751, 1.240239, 1.223499
LPN	0.008425, 0, 0.029901, 0.000203
NAP	0.118168, 0.037538, 0.186183, 0.171212
Overtime, mean, median, SD, IQR	
Combined	0.094551, 0.071222, 0.089417, 0.084389
RN	0.08876, 0.067387, 0.085241, 0.079363
LPN	0.000436, 0, 0.002114, 0
NAP	0.005355, 0.000852, 0.010759, 0.005801
Agency, mean, median, SD, IQR	
Combined	0.087229, 0, 0.244747, 0.031646
RN	0.087178, 0, 0.244623, 0.031646
LPN	3e-06, 0, 7.7e-05, 0
NAP	4.8e-05, 0, 0.000804, 0

© 2025 Pittman P et al. *JAMA Network Open*.

2019

2.621387, 2.276703, 1.28013, 1.20937
2.469921, 2.132294, 1.159978, 1.044088
0.011063, 0, 0.038356, 0
0.140402, 0.062587, 0.210511, 0.202748

2.470099, 2.209129, 1.182273, 1.039658
2.325245, 2.04861, 1.062501, 0.982141
0.010657, 0, 0.037572, 0
0.134197, 0.060508, 0.200173, 0.192478

0.097684, 0.077129, 0.079422, 0.065929
0.091072, 0.071306, 0.072894, 0.059086
0.000406, 0, 0.001564, 0
0.006205, 0.001392, 0.01259, 0.00657

0.053604, 0, 0.187733, 0
0.053604, 0, 0.187733, 0
0, 0, 0, 0
0, 0, 0, 0

2020

2.797263, 2.41243, 1.458958, 1.310788
2.633907, 2.29511, 1.339384, 1.131885
0.0097, 0, 0.033881, 0.000997
0.153656, 0.064086, 0.240255, 0.206572

2.606899, 2.277561, 1.324598, 1.213963
2.450134, 2.153858, 1.201411, 1.039882
0.009272, 0, 0.032916, 0.000945
0.147493, 0.063064, 0.231076, 0.205117

0.106717, 0.08188, 0.089291, 0.078656
0.100133, 0.076419, 0.082214, 0.074281
0.00042, 0, 0.002039, 0
0.006163, 0.001016, 0.013269, 0.00613

0.083647, 0, 0.306617, 0.000321
0.083639, 0, 0.306607, 0.000321
8e-06, 0, 0.000133, 0
0, 0, 0, 0

© 2025 Pittman P et al. *JAMA Network Open.*

2021

1.827888, 1.723155, 1.335484, 1.903055
1.720702, 1.649194, 1.273411, 1.78107
0.006576, 0, 0.023386, 0.000149
0.100611, 0.02019, 0.145402, 0.151119

1.630204, 1.564257, 1.262616, 1.935068
1.527675, 1.517145, 1.198284, 1.902823
0.006042, 0, 0.021085, 0.000123
0.096487, 0.016467, 0.141542, 0.140929

0.090817, 0.06571, 0.095392, 0.103995
0.086189, 0.061991, 0.094041, 0.106743
0.000529, 0, 0.002825, 0
0.004099, 0.000362, 0.006791, 0.005093

0.106884, 0, 0.230781, 0.080766
0.106855, 0, 0.230714, 0.080766
4e-06, 0, 6.9e-05, 0
2.5e-05, 0, 0.000295, 0

2022

1.755691, 1.624391, 1.313505, 1.79663
1.653838, 1.562884, 1.248369, 1.777632
0.007827, 0, 0.02516, 0
0.094026, 0.012063, 0.147434, 0.130443

1.565797, 1.459101, 1.239565, 1.984927
1.46931, 1.438638, 1.171901, 1.907716
0.007442, 0, 0.023647, 0
0.089045, 0.011325, 0.142475, 0.121436

0.080815, 0.053993, 0.09216, 0.084212
0.075632, 0.048634, 0.090342, 0.078409
0.000385, 0, 0.001844, 0
0.004799, 0.000539, 0.008344, 0.00536

0.109079, 0, 0.233803, 0.131191
0.108897, 0, 0.233336, 0.131191
0, 0, 0, 0
0.000183, 0, 0.001659, 0

© 2025 Pittman P et al. *JAMA Network Open.*

eTable 2. Baseline regression model for primary outcomes with healthcare personnel hours per patient day variables in hospitals

	PSI-6		PSI-8		PSI-10		PSI-11	
	Estimate	p-value	Estimate	p-value	Estimate	p-value	Estimate	p-value
**RN/LPN work HPPD**	0.05 (0.09)	0.53	0.17 (0.05)	0.14	0.00 (0.09)	0.97	-0.04 (0.05)	0.37
**RN/LPN overtime HPPD**	0.81 (1.00)	0.42	-1.35 (1.63)	0.4	-0.69 (1.12)	0.53	0.21 (0.57)	0.71
**RN/LPN agency HPPD**	0.21 (0.32)	0.52	-0.72 (0.63)	0.25	-0.32 (0.36)	0.37	0.21 (0.16)	0.2
**NAP work HPPD**	-2.59 (0.47)	0.58	-1.69 (0.77)	0.02	-0.25 (0.42)	0.55	0.13 (0.21)	0.52
**Intercept**	-0.35 (0.22)	0.11	0.14 (0.30)	0.65	0.23 (0.25)	0.35	0.02 (0.16)	0.89
**Covariates**	V		V		V		V	

SOURCE [Premier Inc., Authors analysis] Note: This table displays coefficient estimates from regression models assessing patient safety indicators (PSI-6, PSI-8, PSI-10, and PSI-11). The models include controls for healthcare personnel hours per patient day (HPPD) metrics, including RN/LPN work, RN/LPN overtime, RN/LPN agency, and NAP work HPPD, along with an intercept and additional covariates. HPPD: hours per patient per day. Robust standard errors in parentheses

eTable 3. Baseline regression model for primary outcomes with healthcare personnel hours per patient day variables in hospitals (cont.)

	PSI-12		PSI-13		PSI-14		PSI-15	
	Estimate	p-value	Estimate	p-value	Estimate	p-value	Estimate	p-value
**RN/LPN work HPPD**	0.07 (0.04)	0.09	-0.01 (0.06)	0.93	-0.03 (0.11)	0.77	-0.01 (0.08)	0.87
**RN/LPN overtime HPPD**	-0.56 (0.51)	0.27	-0.42 (0.73)	0.56	-1.97 (1.37)	0.14	-0.49 (0.96)	0.61
**RN/LPN agency HPPD**	0.13 (1.64)	0.42	-0.03 (0.21)	0.87	0.09 (0.43)	0.83	0.21 (0.31)	0.49
**NAP work HPPD**	-0.36 (0.21)	0.08	0.09 (0.26)	0.73	0.26 (0.61)	0.67	-0.29 (0.43)	0.49
**Intercept**	-0.47 (0.11)	<.001	-0.17 (0.18)	0.35	-0.09 (0.28)	0.75	-0.18 (0.21)	0.4
**Covariates**	V		V		V		V	

SOURCE [Premier Inc., Authors analysis] Note: This table displays coefficient estimates from regression models assessing patient safety indicators (PSI-12, PSI-13, PSI-14, and PSI-15). The models include controls for healthcare personnel hours per patient day (HPPD) metrics, including RN/LPN work, RN/LPN overtime, RN/LPN agency, and NAP work HPPD, along with an intercept and additional covariates. HPPD: hours per patient per day. Robust standard errors in parentheses

© 2025 Pittman P et al. *JAMA Network Open.*

eFigure 1. Non-linear regression model outcomes for RN/LPN overtime HPPD on the PSI-03 in hospitals

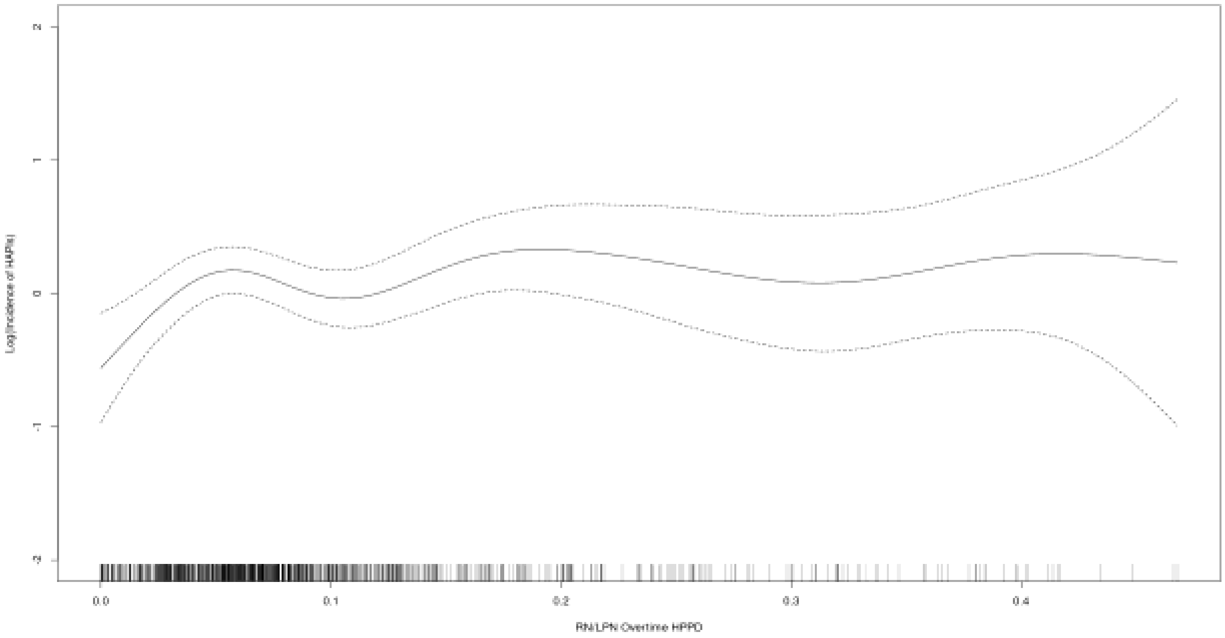

Note/Source: SOURCE [Premier Inc., Authors analysis] Note: This graph shows the association between RN/LPN overtime HPPD and PSI-03. Below a threshold of 0.032 HPPD, a lower risk of PSI-03 was identified. However, surpassing this threshold, additional overtime HPPD was significantly correlated with an increased risk of PSI-03 (B-spline $p < 0.001$).

© 2025 Pittman P et al. *JAMA Network Open*.

eFigure 2. Non-linear regression model outcomes for RN/LPN agency HPPD on PSI-03 in hospitals

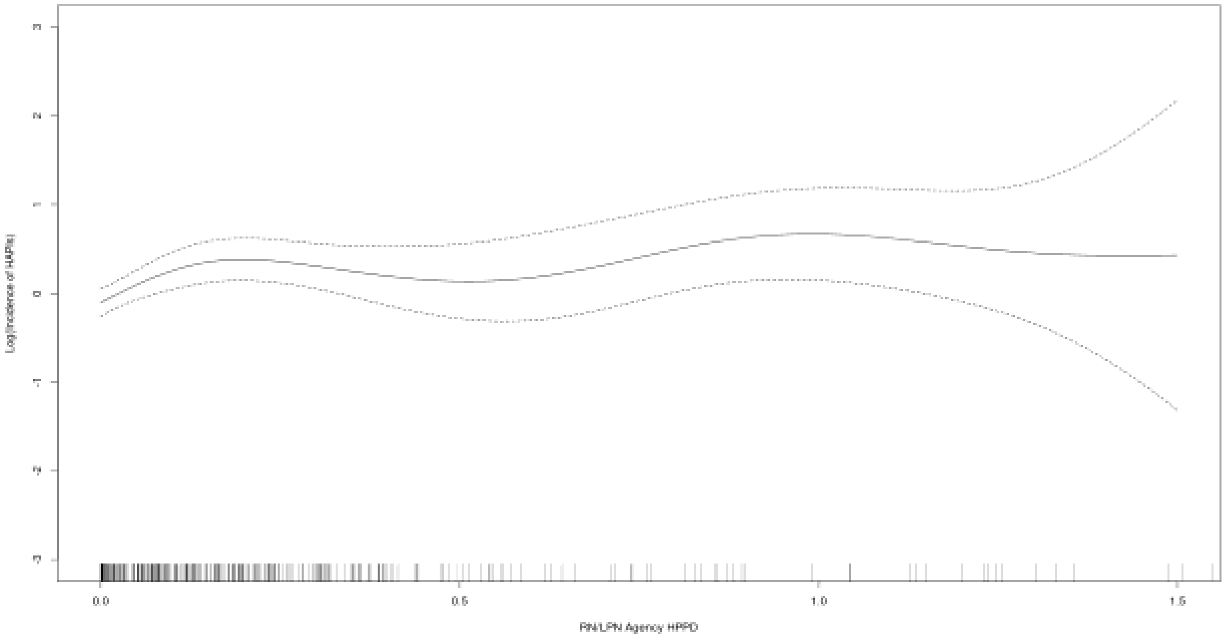

Note/Source: SOURCE [Premier Inc., Authors analysis] Note: This graph shows the association between RN/LPN agency HPPD and PSI-03. Below the threshold of 0.028 HPPD, a lower risk of PSI-03 was evident. However, upon surpassing this threshold, there was a notable increase in the risk of PSI-03 (B-spline $p < 0.001$).

© 2025 Pittman P et al. *JAMA Network Open*.
