# Supplementary material for: Increased Utilization of Overtime and Agency Nurses and Patient Safety
Source: JAMA Netw Open. 2025 Apr 2;8(4):e252875. doi: 10.1001/jamanetworkopen.2025.2875 (PMC11966309; doi:10.1001/jamanetworkopen.2025.2875)
Supplement: Supplement 2. — Data Sharing Statement [file jamanetwopen-e252875-s002.pdf]

Data Sharing Statement

Pittman. Increased Utilization of Overtime and Agency Nurses and Patient Safety. *JAMA Netw Open*. Published April 02, 2025. doi:10.1001/jamanetworkopen.2025.2875

Data
Data available: No
